# Effect of Salt Concentration on Oil Recovery during Polymer Flooding: Simulation Studies on Xanthan Gum and Gum Arabic

**DOI:** 10.3390/polym15194013

**Published:** 2023-10-07

**Authors:** Oluwasanmi Olabode, Oluwatimilehin Akinsanya, Olakunle Daramola, Akinleye Sowunmi, Charles Osakwe, Sarah Benjamin, Ifeanyi Samuel

**Affiliations:** Department of Petroleum Engineering, Covenant University, Ota 112104, Nigeria; akinsanya.oluwatimilehin@stu.cu.edu.ng (O.A.); olakunle.daramola@covenantuniversity.edu.ng (O.D.); chukwudalu.osakwe@stu.cu.edu.ng (C.O.);

**Keywords:** polymer (xanthan and guar gums), salt concentration, reservoir simulation, enhanced oil recovery

## Abstract

Oil recoveries from medium and heavy oil reservoirs under natural recovery production are small because of the high viscosity of the oil. Normal water flooding procedures are usually ineffective, as the injected water bypasses much of the oil because of its high mobility. Thermal flooding processes are desirable but have many disadvantages from costs, effects on the environment, and loss of lighter hydrocarbons. Chemical flooding options, such as bio-polymer flooding options, are attractive, as they are environmentally friendly and relatively cheap to deploy and help to increase the viscosity of the injecting fluid, thereby reducing its mobility and increasing its oil recovery. The downside to polymer flooding includes reservoir temperature, salinity, molecular weight, and composition. Six weight percentages of two polymers (xanthan gum, XG, and gum arabic, GA) are dissolved in water, and their viscosity is measured in the laboratory. These viscosities are incorporated with correlations in the Eclipse software to create models with different polymer concentrations of (0.1% wt., 0.2% wt., 0.3% wt., 0.4% wt., 0.5% wt., and 1% wt.). A base case of natural recovery and water injection was simulated to produce an oil recovery of 5.9% and 30.8%, respectively, while at 0.1% wt. and 1% wt., respectively, oil recoveries of 38.8% and 45.7% (for GA) and 48.1% and 49.8% (for XG) are estimated. At 5% and 10% saline conditions, a drop in oil recovery of (4.6% and 5.3%) is estimated during GA flooding and (1.2% and 1.7%) for XG flooding at 1% wt., respectively. XG exhibits higher oil recoveries compared to GA at the same % wt., while oil recoveries during GA floodings are more negatively affected by higher saline concentrations.

## 1. Introduction

An essential property for optimized oil production is its API gravity or fluid density. Reservoirs producing medium to heavy crude oil or those with an API gravity range of between 22.3 and 31.1° for medium crude oil and 10 and 22.3° [1] for heavy crude pose serious production challenges. Naturally, heavy crude oil will not flow because of its viscous nature, and to optimize production, secondary or enhanced recovery processes need to be implemented [2]. The option for water flooding is a natural selection for the optimization of oil production. However, the strategy is ineffective as the density of the injected water is lower than the residual heavy oil. This often leads to an irregular flood pattern that is enhanced by rock matrixes, well patterns, reservoir heterogeneities, and anisotropy [3]. Options for enhanced oil recovery are mostly screened and ranked based on criteria relating to reservoir fluids and rock properties, costs, and reserves [4]. Field implementations for thermal-enhanced oil recovery are challenging because of cost, loss of heat, the effect of heat signatures on the environment, coke formation, and loss of the lighter components of the hydrocarbon (for in situ combustion processes), and the scaling and fouling of facilities [5]. A polymer-enhanced oil recovery is a suitable option for reservoirs producing heavy oil; when added to the injection water, they reduce its mobility and increase its viscosity, enabling a uniform and favorable oil displacement. To minimize costs, certain biopolymers can be sourced from waste materials like solanum tuberosum and banana peels [6] to be utilized as an EOR option for heavy oil reservoirs.

Xanthan gum (XG) is an anionic biopolymer with repeated chains of cellulose monosaccharides and oligosaccharides. One of the key characteristics of xanthan gum is its high viscosity combined with a very high pseudo-plasticity, which means that its apparent viscosity decreases with an increase in applied shear force. In addition, XG is stable over a wide range of temperatures and pH; as well, it is water soluble but insoluble in a wide range of organic solvents. The rheology of aqueous solutions of XG has been studied over a wide range of shear rates and concentrations. At sufficient dilution and low shear rates, xanthan solutions show a region of Newtonian viscosity behavior [7]. Gum arabic, a polyelectrolyte, is an emulsifier with binding, stabilizing, and shelf-life-enhancing properties. Gum arabic (GA) is a low-viscosifying enhancement agent and has a low molecular weight, unlike other biopolymers, and has an oil–brine IFT reduction capacity [8,9]. Its capacity to reduce oil–brine IFT is related to the polymer’s ability to migrate to the oil–water interface, in which the hydrophobic polypeptide chain interacts with oil and the hydrophilic arabinogalactan unit interacts with water [10]. It should be noted that its surface activity properties are weak compared to most other surfactants. References [9,11,12], in their studies, have summarized factors that affect the performance of polymers during enhanced oil-recovery processes. These factors include reservoir temperature, molecular weight, the composition of the polymer, the salt concentration of the polymer, and reservoir fluid. Other factors might include the injection rates, number of wells, and reservoir heterogeneity, which affects the adsorption rates of polymers on rock surfaces. Salts like sodium chloride (N_a_Cl), when present at concentrations as low as 2% wt., can result in a 23.8% loss in viscosity, while a similar concentration of divalent ions (Ca^2+^) resulted in an approximately 28.6% loss of viscosity [13]. The presence of salt causes an electrostatic repulsion of polymer chains, which leads to its contraction and the eventual loss of viscosity [14]. Simulation studies on low-salinity-water flooding have been considered by [15] to reduce the reservoir saline contents for light oil reservoirs with additional recoveries of 11% over water flooding, but the challenge with this option is low performance in medium to heavy oil reservoirs [16]. References [17,18,19] have experimentally investigated the oil recovery performances of polymer flooding options with a preconditioned reservoir with low salinity. Most studies on EOR (enhanced oil recovery) have been conducted via experimental studies. This involves obtaining representative samples of core samples from reservoirs (which must be preserved) and crude oil samples and the availability of permeability testers for core flooding processes. This can be time consuming and coupled with errors that can occur during the measurements. Hence, a simulation study can be conducted using software to carry out the experimental operation. A reservoir model can be built and incorporated with experimental data (such as polymer viscosities, shear rates, salt concentrations, etc.) using designated keywords. The software also offers similar examples and workflow guides/instructions to carry out polymer flooding. A simulation study will also allow for multiple-sensitivity analysis to be carried out effectively, as compared to core flooding experiments where core samples must be recycled/desaturated to subject them to new experimentation processes. The desaturation processes are not always efficient; thus, residues of previous experiments can alter the results of the next set of experiments if used. Another challenge with the experimental option (especially pore/core options), aside from the cost and duration, is the approach in carrying out multiple sensitivities to properly investigate the effect of salt on oil recoveries during polymer flooding. Furthermore, a preconditioned saline medium creates a static condition that makes the estimation of oil recoveries during saline changes problematic. This study first considers the effect of six different concentrations of xanthan and gum arabic polymers on oil recovery from a heavy oil reservoir created using the Eclipse black oil model. In doing so, models based on specified concentrations are developed with similar rock and fluid properties, fluid contacts, and well designs. Each of these models is subsequently subjected to two concentrations of the saline reservoir conditions. It is important to note that this is not a low-salinity flooding procedure but a natural saline condition of the reservoir fluids/injected polymers.

## 2. Methodology

### 2.1. Rheological Measurements

**Figure 1:**
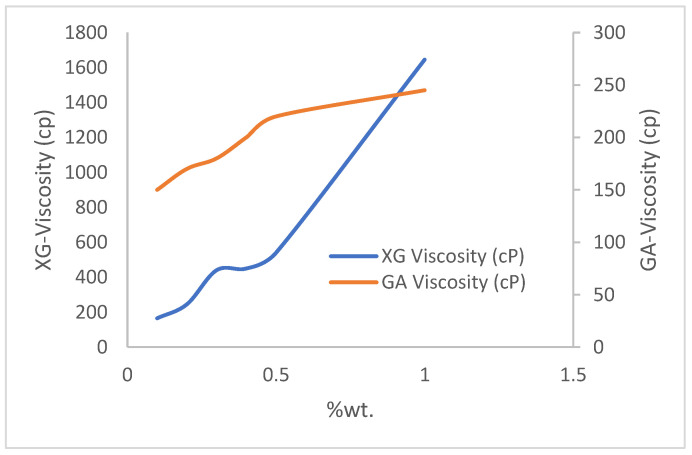
Viscosity plot.

**Figure 2:**
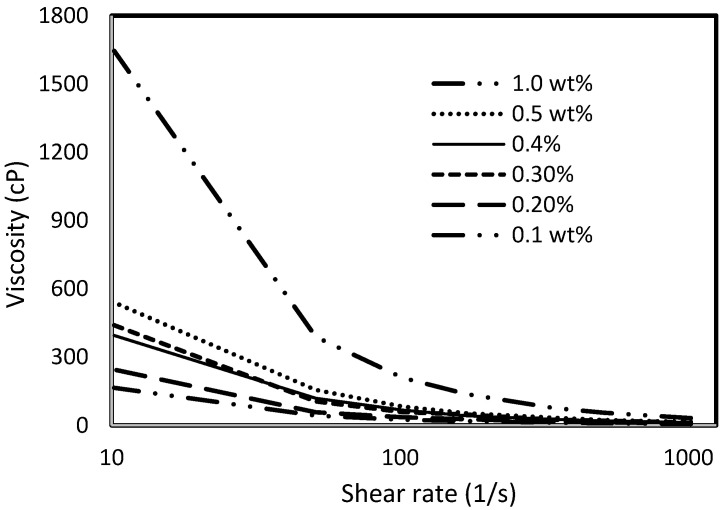
Viscosity vs. shear rate profile for XG.

**Figure 3:**
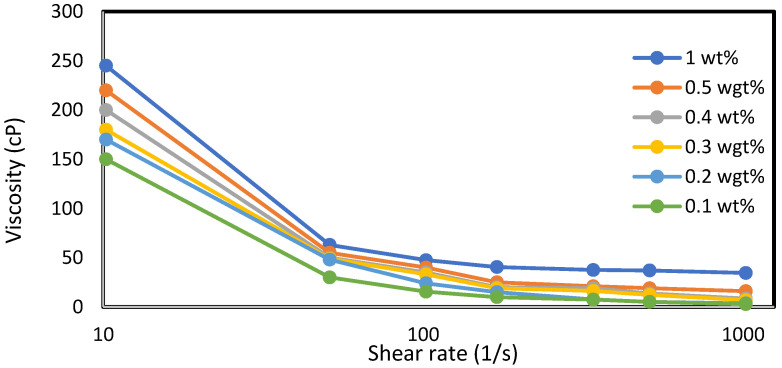
Viscosity vs. shear rate profile for GA.

Six weight percentages (0.1% wt., 0.2% wt., 0.3% wt., 0.4% wt., 0.5% wt., and 1% wt.) of xanthan and guar gums are measured using a conventional top-loading balance (OASIS 2A) and dissolved in 200 mL (0.001729698 stb) of deionized water. The viscosities of the resulting mixtures are measured using an OFITE 800 viscometer (manufactured by OFITE, Houston TX, USA). Figure 1 shows the viscosity trend per weight concentration of the polymers. This trend is also supported by the experimental studies of [20]. They concluded that increasing the weight-percent concentration of the polymer increases the contacts between the molecules and, hence, results in an increase in viscosity. The large difference in viscosity experienced at 30 °C between the xanthan and gum arabic is attributed to the differences in their molecular weights. References [21,22,23] have studied the molecular structures and weights of xanthan and gum arabic and found them to be in the range of 2 × 10^6^ to 20 × 10^6^ and 2.5 × 10^5^ to 1 × 10^6^ Daltons, respectively: an indication that the viscosity of XG at 1% weight is approximately nine times that of GA (which is illustrated in Figure 1). The behavior of the mixtures under the application of stress is represented by viscosity-versus-shear-stress profiles in Figure 2 and Figure 3 for xanthan and gum arabic, respectively. These figures indicate an inverse relationship between the polymer solutions and the corresponding shear rates. The data for these figures are highlighted in Appendix A. The more viscous a polymer sample (i.e., 1% wt.), the lower the rate of its deformity because of the higher strength between the polymer bonds at higher concentrations. Thus, as the shear rates increase, the viscosity of the polymer solution reduces. This reduction is more drastic for lower polymer concentrations (Figure 3). Due to the marginal differences in GA viscosity (Figure 1), the decline in shear rate observed follows a marginal trend (Figure 2), resulting in lower viscosity values at higher shear rates.

### 2.2. Reservoir Modeling

**Figure 4:**
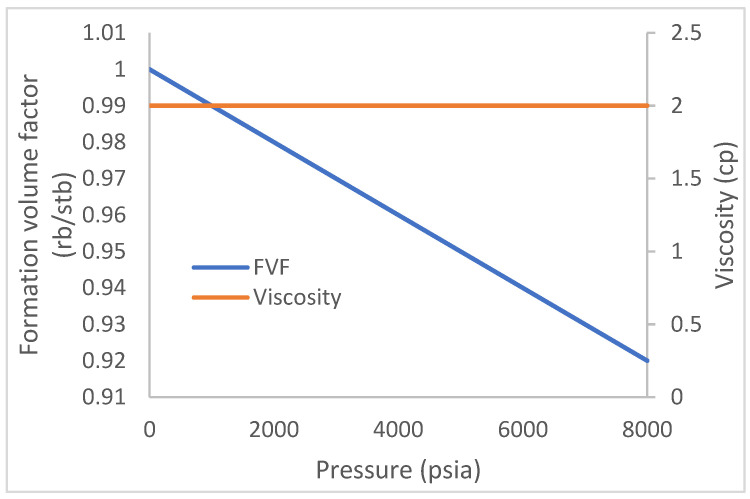
Oil property (with no dissolved gas).

The Schlumberger reservoir simulator (ECLIPSE) is used to create a reservoir model with a 10 by 10 by 1 number of cells in the X, Y, and Z coordinates. The model is initialized with oil and water at the RUNSPECP section of the simulator. The field unit option is selected at a simulation start date of 15th November 2021 and a cumulative production time of 1700 days. In the grid section, a porosity value of 0.2 is indicated for all 100 cells, while a permeability of 1500 md is initiated for both the X and Y directions. This initializes the model as one with homogeneous properties. The depths to the TOPs faces are 4000 ft, while the sizes of each cell in the X, Y, and Z directions are 75 ft, 75 ft, and 30 ft, respectively. The PVT section describes the properties of the initiated reservoir fluids and foam properties using certain keywords, as described in the following section. The fluid densities in lb/ft^3^ for oil, water, and gas are 58, 45, and 0.044, respectively. This is at a reference pressure of 4000 psia and a rock/water compressibility of 4 × 10^−6^/psi and 3 × 10^−6^/psi. The water formation volume factor and viscosity used are 1 rb/stb and 0.5 cp, respectively. The oil property in Figure 4 shows the relationship (of oil without dissolved gas) that exists between the pressure, formation volume factor in rb/stb, and oil viscosity. Due to the absence of dissolved gases, the oil viscosity remains constant. There is a reduction in reservoir/fluid pressure during the production of oil from the reservoir to the surface production facilities. This reduction should normally result in a phase change with an evolution of gases from the oil. The evolution of gas from oil normally heralds a reduction in oil production, but Figure 4 describes a low reduction in oil formation (0.08 rb/stb) over an 8000 psi drop in pressure. This is to show that little or no gas was liberated, and the viscosity of the oil remained the same throughout the pressure drop period.

Twelve polymer concentrations (six for XG and six for GA) are developed to create ten different models. The models are first initialized with polymer functions like the PLYVIC (Table 1), which is the polymer viscosity function in the absence of salt and a PLYMAX, which indicates an initial value of zero salt concentration. To indicate the presence of salt in the polymer solution, the keyword PLYVICS (Table 2) is introduced to represent the polymer viscosity function in the presence of salt. Two salt concentrations of 5% wt. and 10% wt. are considered for each polymer concentration of GA and XG, respectively.

**Figure 5:**
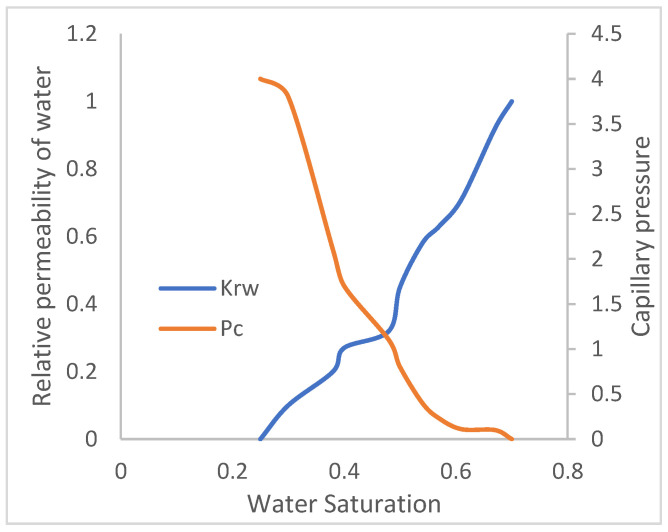
Water-relative permeability.

**Figure 6:**
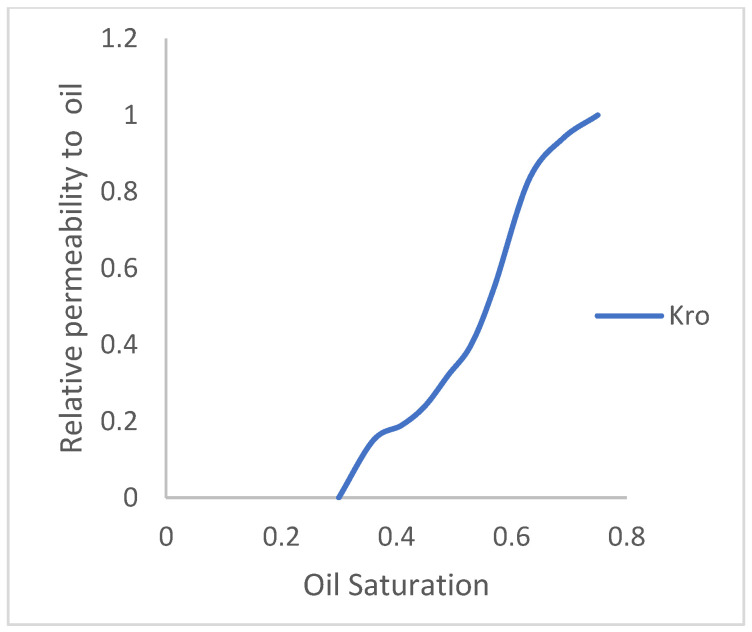
Oil-relative permeability.

The reservoir model has two fluids (oil and water). In a reservoir medium, one fluid will flow relative to another and vice versa. Permeability is denoted as K, and it is the ability of a fluid to flow. Thus, Krw is the relative permeability of water to flow in the presence of oil, and Kro is the relative permeability of oil to flow in the presence of water, while Pc is the capillary pressure acting in the reservoir medium that prevents the dominance of flow of one fluid over the other. The water and oil saturation functions, as they relate to their respective relative permeability and capillary pressures, are described in Figure 5 and Figure 6. As the permeability or tendency of each fluid to flow increases, its saturation reduces.

The polymer adsorption (PLYADS) property that describes the rate of polymer adoption or retention on rock surface in the presence of 5% and 10% is assumed to be at a factor of 0.09 lb/lb and 0.11 lb/lb (for GA and XG, respectively), as it is expected that the polymer concentration is not a determining factor on the rate of polymer absorption. The initial polymer adsorption at zero polymer concentration is defaulted to zero. The polymer rock property PLYROCK (Table 3) specifies the rock properties for the polymer rock model for both salt models. It describes the relationship between the dead pore spaces, residual resistant factor, the mass of rock, adsorption index, and maximum adsorption value.

The keyword EQUIL is used to specify a datum depth of 4000 ft, pressure at a datum depth of 4000 psia, and a water–oil contact of 6000 ft, giving a pay thickness of 2000 ft. The salt concentrations vs. depths for both salt concentrations are shown in Table 4. The initial water concentration is indicated by SWAT at 0.25 and the initial pressure by PRESSURE at 4000 psia. The initial salt concentration for the two salt models is given by 100 * 10 and 100 * 5, where 100 is the total number of cells, while 5 and 10 are the salt concentrations. The model is initialized based on the water and gas saturations, the fluid contacts, and the pressures (rock and fluid) to obtain the initial fluids in place of 470,472 stb and 152,114 stb of oil and water, respectively.

The schedule sections help in creating wells and assigning functions to them. The keyword WELSPECS is used to create wells, a producer well (P) and an injector well (I), with the coordinates described in Table 5, while Table 6 describes the well connection within the reservoir.

The keyword WCONPROD is used to indicate an oil production rate of 1000 stb/day, while a water injection rate of 200 stb/day is indicated by WCONINJ. The keyword WPOLYMER is used to indicate the respective wells’ polymer concentrations. Table 7 describes these concentrations for the case studies of the two salt concentration models and the normal polymer model.

The following case studies are considered in this study:

1. Natural production from the polymer model;
2. Water injection;
3. Production from polymer injection at specified concentrations in Table 1;
4. Production from polymer injection at specified concentrations from Table 7 at salt concentrations of 5% and 10%.

The summary section includes all the output variables that will be used in this study for comparing the efficiencies of the case studies listed above. These variables include FOE (oil recovery factor), WOPT (well oil production total), WWCT (well water cut), and WOPR (well oil production rate).

## 3. Results

The results highlight the input vectors in the summary section of the software for the case studies. The highlight will be based on a comparison using plots and bar charts.

### 3.1. Case 1: Natural Production and Water Injection

**Figure 7:**
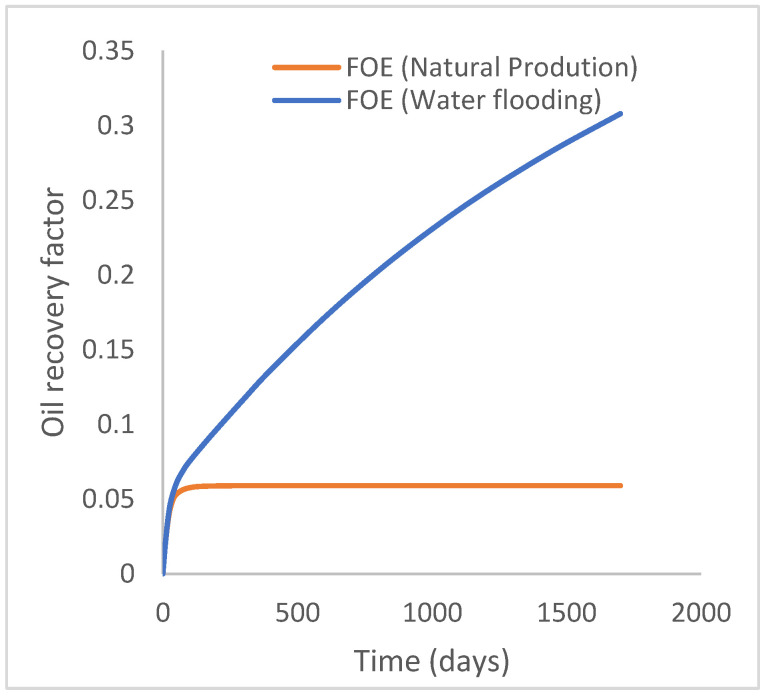
Oil recovery.

**Figure 8:**
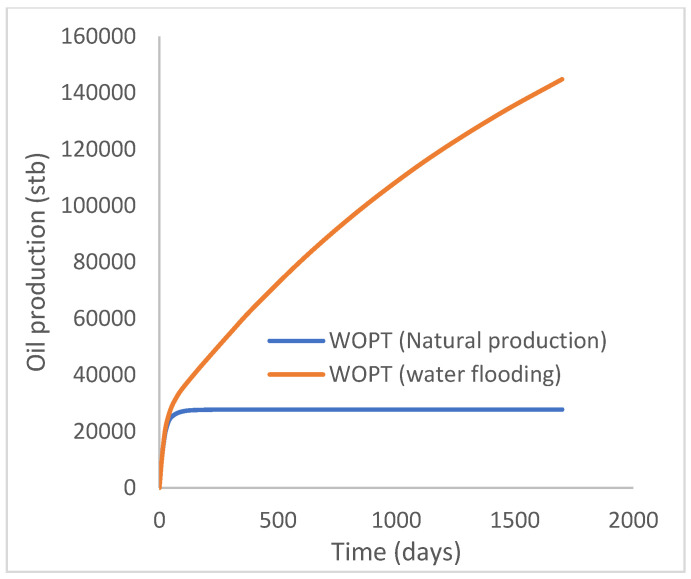
Oil production.

**Figure 9:**
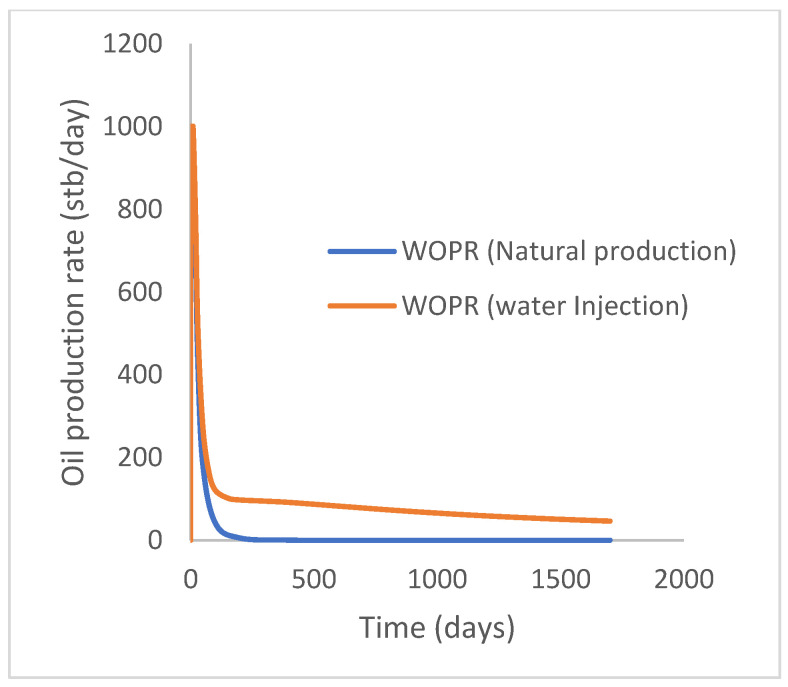
Oil production rate.

**Figure 10:**
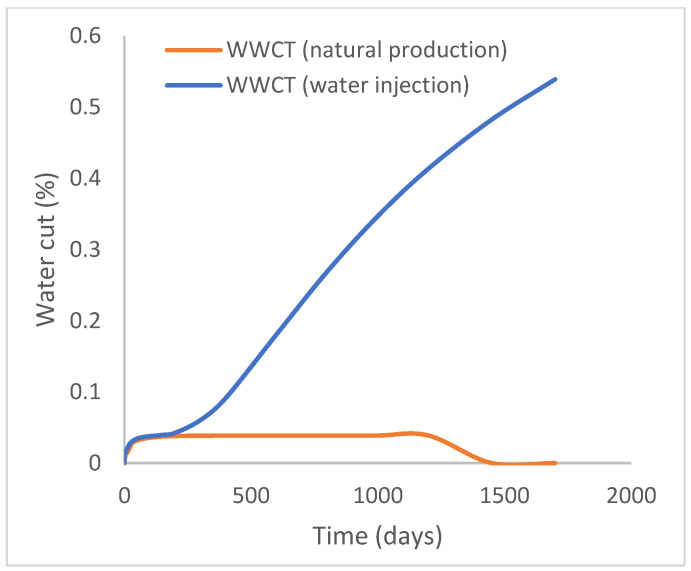
Water cut.

**Figure 11:**
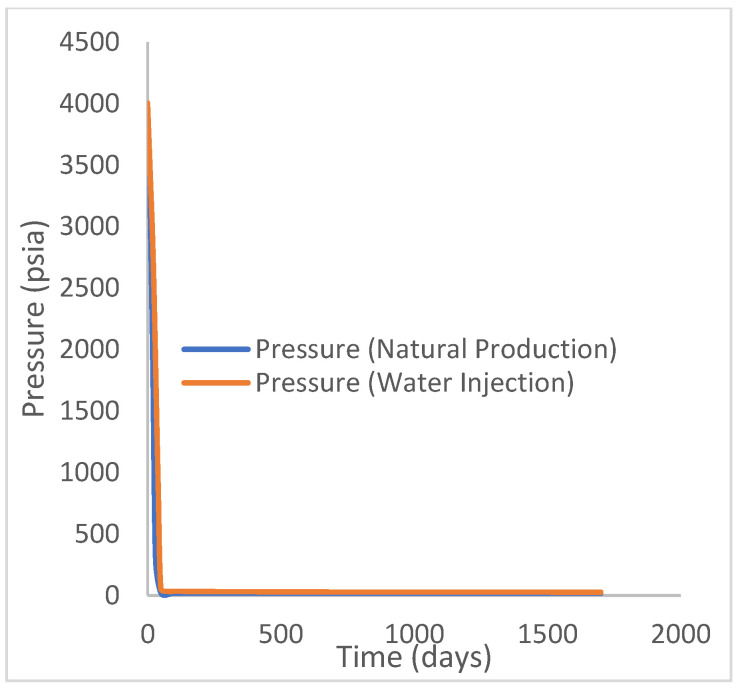
Field pressure.

This scenario case describes the natural production from the well using both the reservoir energy and water injection processes. The injector well has been initiated but has been shut in during the natural injection, while the injector well is initiated with the water injection option at 200 stb/day. Figure 7 shows the oil recovery at 5.90% and 30.78% during natural and water flooding production scenarios, respectively; this gives an incremental oil recovery of 24.88% at a cumulative oil production of 28 Mstb and 145 Mstb, respectively (Figure 8). The oil production rates recorded in Figure 9 show a substantial increase/rise in oil production rates of about 102 stb/day during water injection over natural production. This increase also accounts for the substantial oil recovered and produced during water flooding (Figure 7 and Figure 8). As expected, the water cut during water injection will be higher (Figure 10), reaching a high level of 54%, indicating that most of the injected water has been produced. The plateau trend in Figure 7 and Figure 8 for the natural profiles after almost 30 days of production is an indication of a rapid drop in pressure (Figure 11) at the same interval because of the viscous nature of the oil making it unproduceable from that time onward. Oil production and recovery during water flooding would have similarly taken the same trend as natural production, but the effect of the injected water was felt after 30 days—hence, a rise in oil recovery and production.

### 3.2. Case 2a: Production from GA Polymer Flooding at Different Concentrations

**Figure 12:**
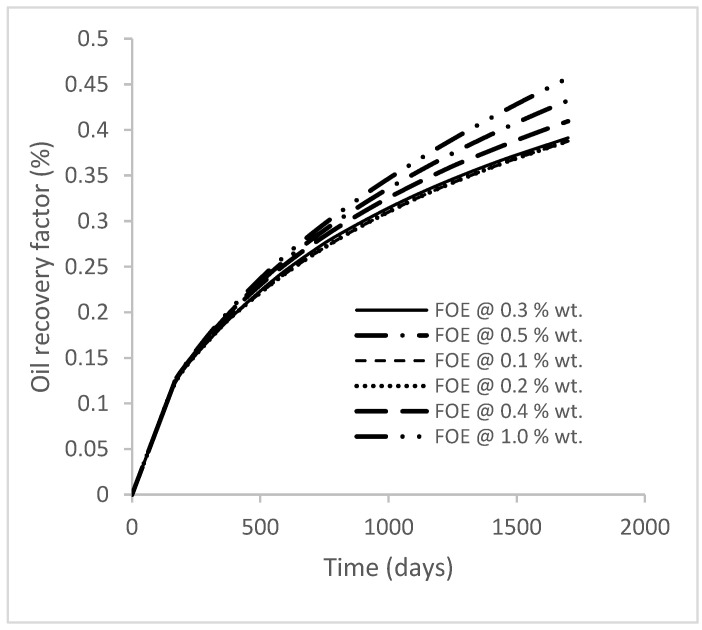
Oil recovery.

**Figure 13:**
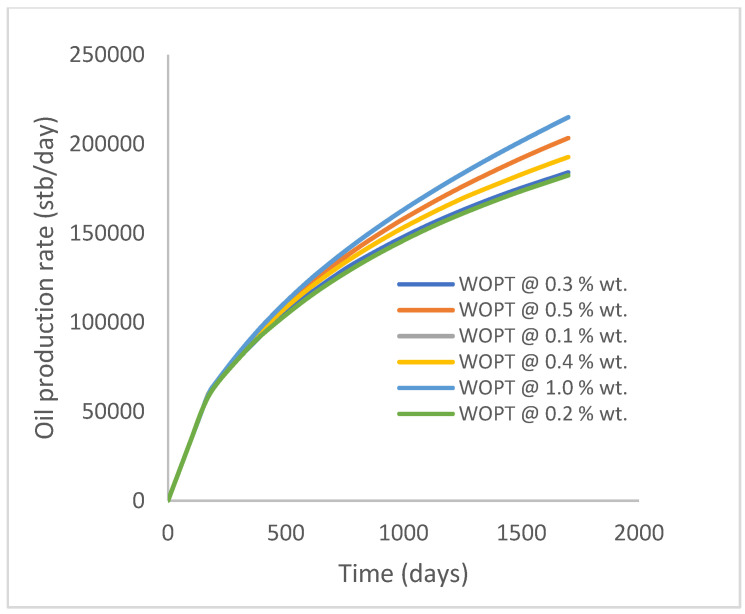
Oil production.

**Figure 14:**
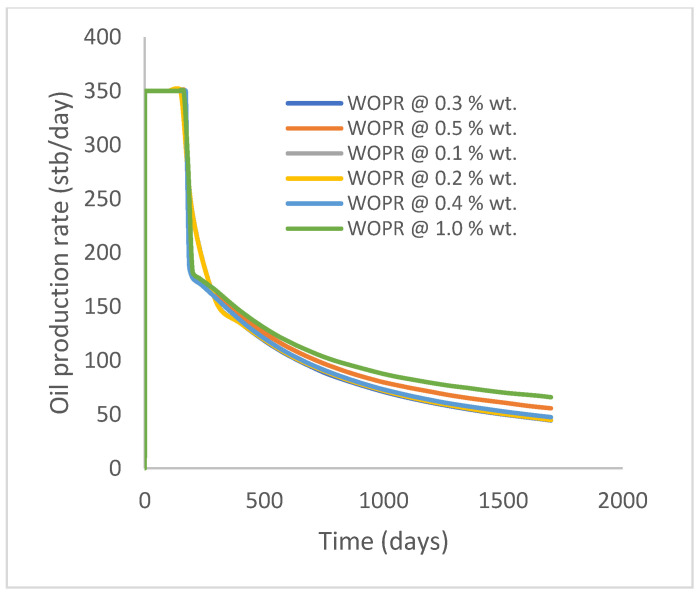
Oil production rate.

**Figure 15:**
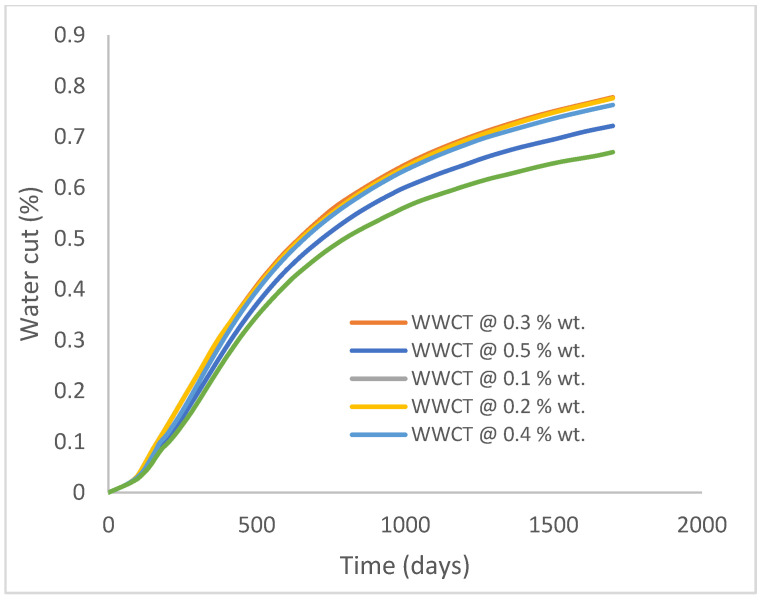
Water cut.

In this case study, the injector well has been initiated with the keyword WPOLYMER to indicate the onset of polymer injection. Figure 12 shows the oil recovery from the six different polymer concentrations. Peak and low oil recoveries of 45.7% and 38.8%, respectively, are recorded under polymer injections at 1.0% wt. and 0.1% wt. and at a cumulative oil production of 215 Mstb and 182 Mstb, respectively (Figure 13). This is an indication that increasing the polymer concentration will increase the oil recovery (Figure 12) and oil production (Figure 13). Each polymer concentration shows a similar increasing trend (Figure 12 and Figure 13) until 162 days before the effect of each % wt. of polymer is felt. A drastic drop in oil production from 350 stb/day to around 60 stb/day is noticeable for all the polymer concentrations (Figure 14). The water cut trends indicate higher water cuts during polymer injections at lower concentrations (Figure 15). The reservoir has two fluids, water and oil; thus, if more oil is produced, less water will be produced, and vice versa. This is also evident in the oil rate (Figure 14), which plateaued for the same 162 days before a dip and a gradual decline.

### 3.3. Case 2b: Production from XG Polymer Flooding at Different Concentrations

**Figure 16:**
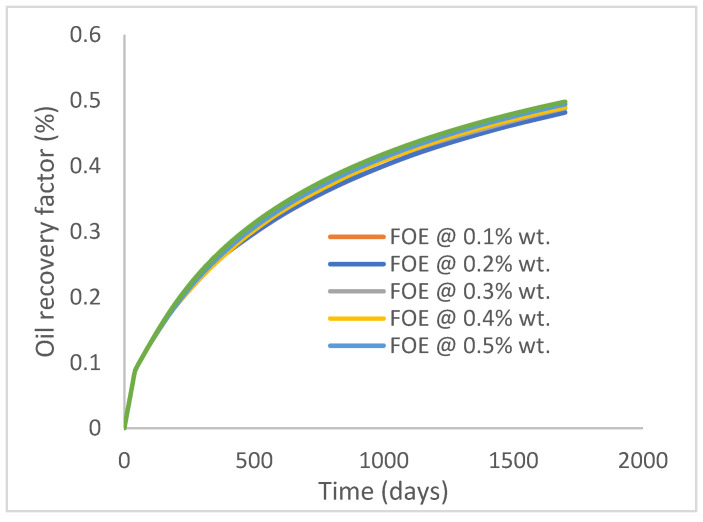
Oil recovery.

**Figure 17:**
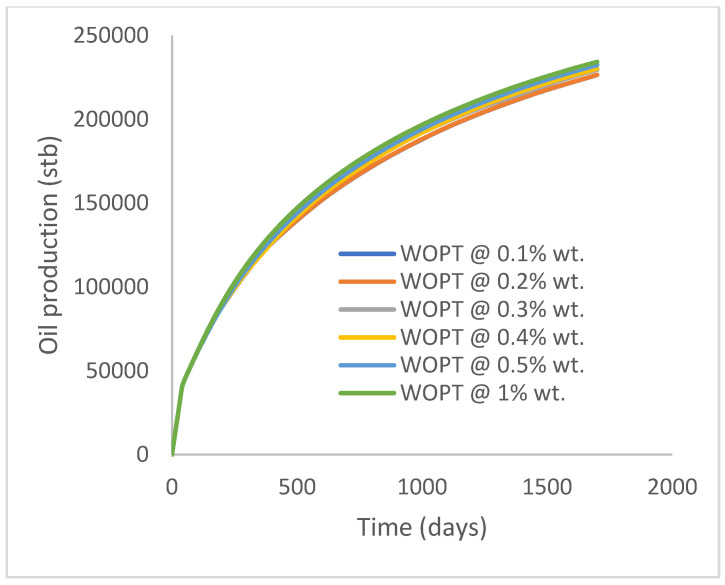
Oil production.

**Figure 18:**
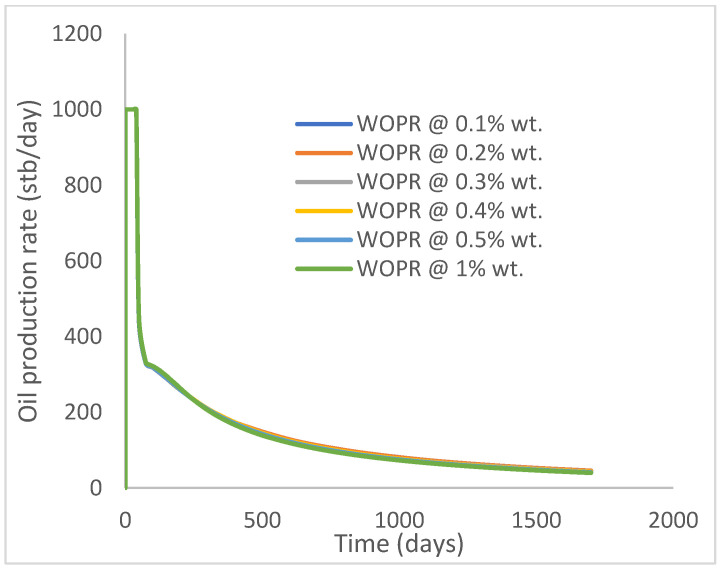
Oil production rate.

**Figure 19:**
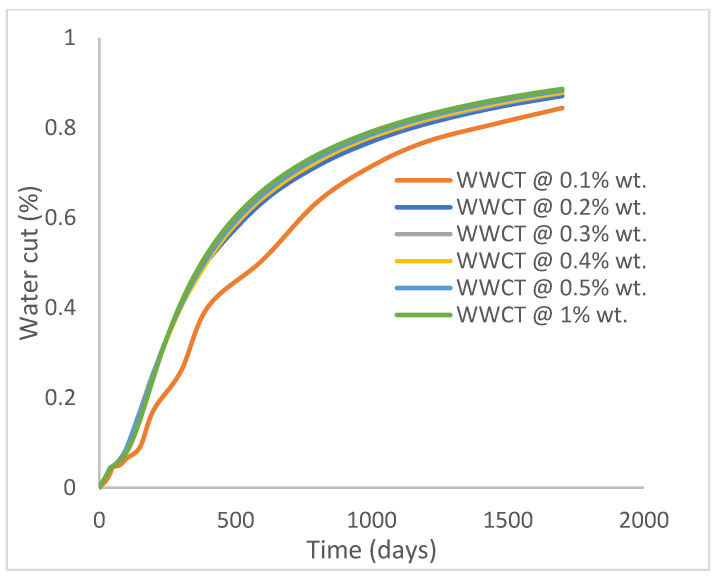
Water cut.

The same keyword is used to initialize polymer injection via the injector well. The oil recovery from the six different polymer concentrations is shown in Figure 16. Peak and low oil recoveries of 49.7% and 48.14% are obtained, respectively, at 1.0% wt. and 0.1% wt., with a total oil output of 234.2 Mstb and 226.5 Mstb (Figure 17). The improved viscosities for each XG % wt. (Figure 2 and Figure 3) resulted in higher recoveries over GA, although the individual differences in the recoveries under this case scenario are not more than 1.64% (considering 1% wt. and 0.1% wt.). An indication of this is that increasing the polymer concentration will not substantially increase oil recoveries, as in the case of GA. A drastic reduction in the oil production rate from 1000 stb/day to around 328 stb/day is noticeable for all the polymer concentrations (Figure 18). The water cut trends indicate higher water cuts during polymer injections at lower concentrations (Figure 19). The reservoir has two fluids, water and oil; thus, if more oil is produced, less water will be produced, and vice versa. A drastic reduction in the oil production rate from 1000 stb/day to around 328 stb/day is noticeable for all of the polymer concentrations (Figure 18). The water cut trends indicate higher water cuts during polymer injections at lower concentrations (Figure 19). The reservoir has two fluids, water and oil; thus, if more oil is produced, less water will be produced, and vice versa.

### 3.4. Case 3a: GA Polymer Injection at 5% wt. Salt

**Figure 20:**
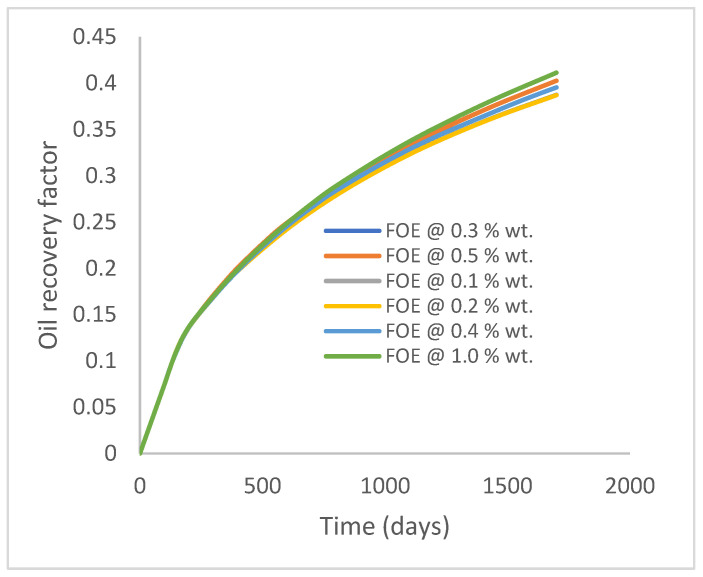
Oil recovery.

**Figure 21:**
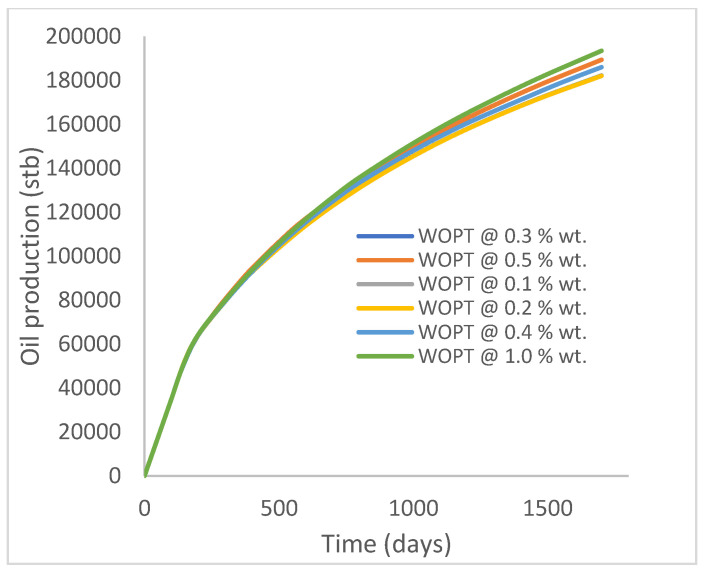
Oil production.

**Figure 22:**
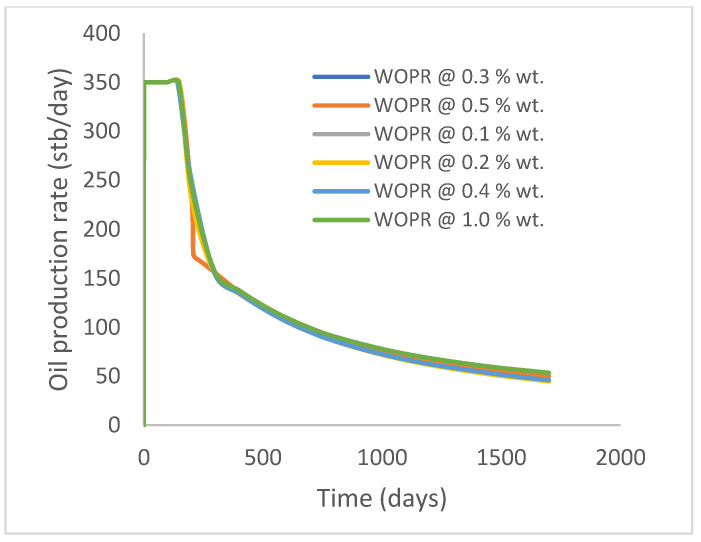
Oil production rate.

**Figure 23:**
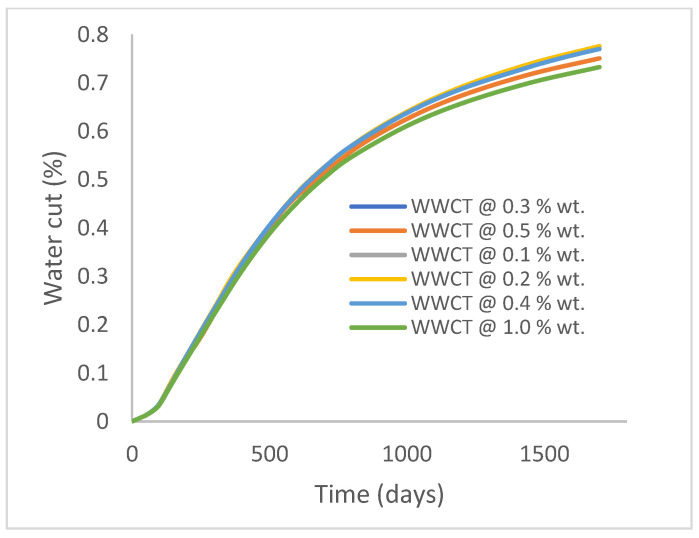
Water cut.

The salt variable in the keyword WPOLMER is initiated to accommodate the initiation of the salt concentration in each polymer concentration subjected to the injector well. The results show a drop in oil recovery at all concentrations compared to case 2a. Oil recoveries of 41.1% and 38.7% are recorded with reference to 1.0% wt. and 0.1% wt. polymers, respectively (Figure 20). This amounts to 4.6% and 0.1% reductions in oil recovery compared to case 2a. The cumulative oil produced at the reference polymer concentrations is in the amounts of 193 Mstb and 182 Mstb, respectively (Figure 21). The summary of the observed oil production rates showed a plateau at 350 stb/day for 146 days before a rapid decline to 156 stb/day after 300 days and, then, a gradual decline in oil production (Figure 22). The effect of close oil production profiles is evident in the water cuts estimated at around 74% (Figure 23).

### 3.5. Case 3b: XG Polymer Injection at 5% wt. Salt

**Figure 24:**
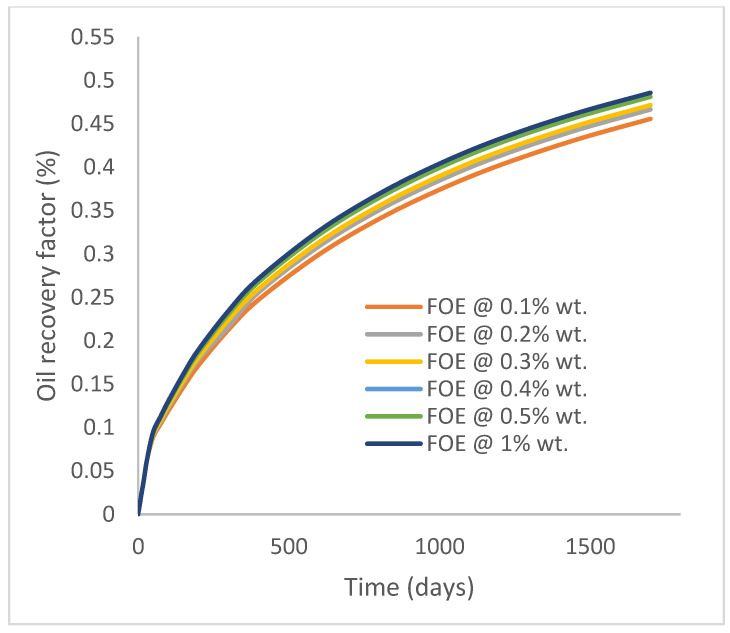
Oil recovery.

**Figure 25:**
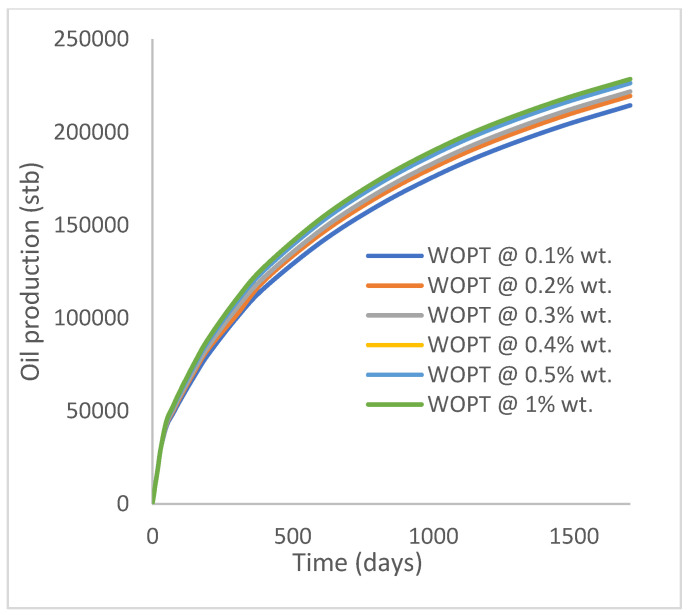
Oil production.

**Figure 26:**
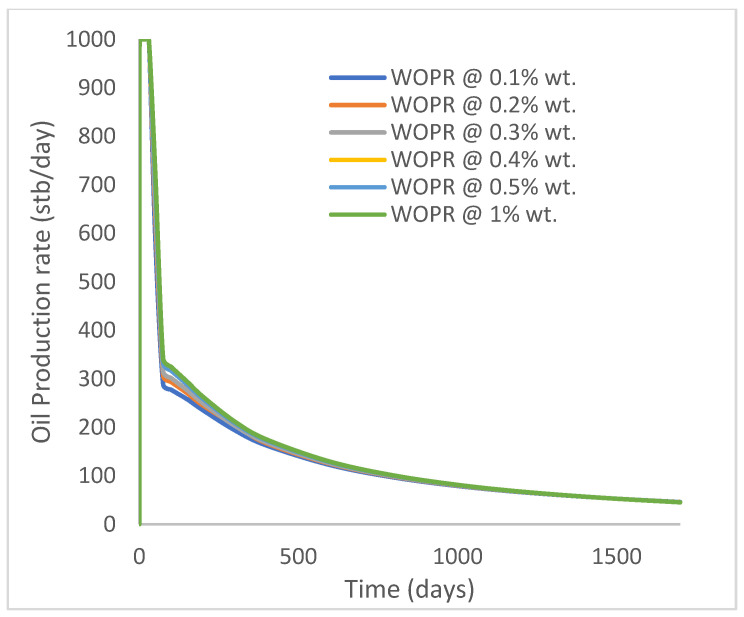
Oil production rate.

**Figure 27:**
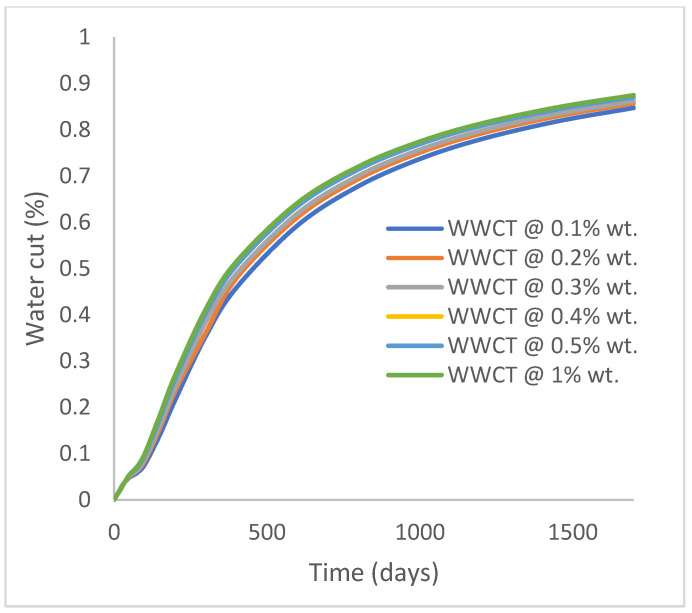
Water cut.

At a salt concentration of 5 wt.%, the findings reveal that the amounts of oil recovered at polymer concentrations of 1.0 wt% and 0.1 wt% are 48.6% and 45.6%, respectively (Figure 24). This shows a decrease of 1.2% and 2.6%, respectively, compared to the results that were reported when salt was not introduced (case 2b), an indication that the presence of salt adversely affects oil recovery and production. The cumulative amounts of oil produced at these salt and polymer concentrations are 228 Mstb and 222 Mstb, respectively (Figure 25), and these oil production estimates have dropped by 6000 stb and 13000 stb, respectively, compared with case 2b. The observed oil production rates show a decline from 1000 stb/day to 323 stb/day after 30 days of constant production rates (Figure 26). Higher water cuts at an average estimate of 85% are observed as cumulative oil production drops (Figure 27).

### 3.6. Case 4a: GA Polymer Injection at 10% wt. Salt

**Figure 28:**
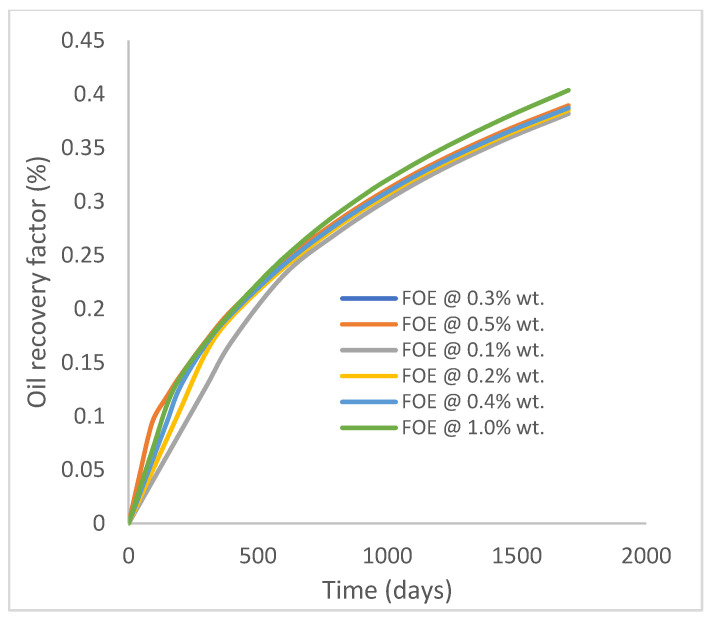
Oil recovery.

**Figure 29:**
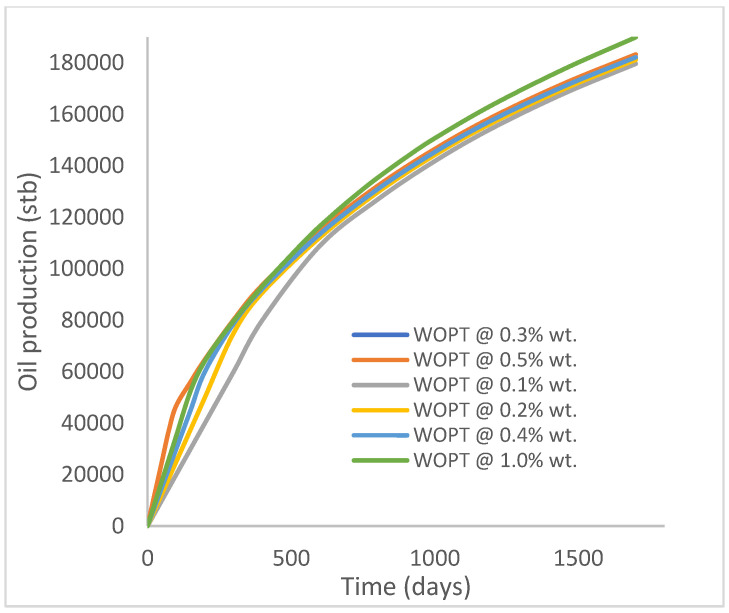
Oil production.

**Figure 30:**
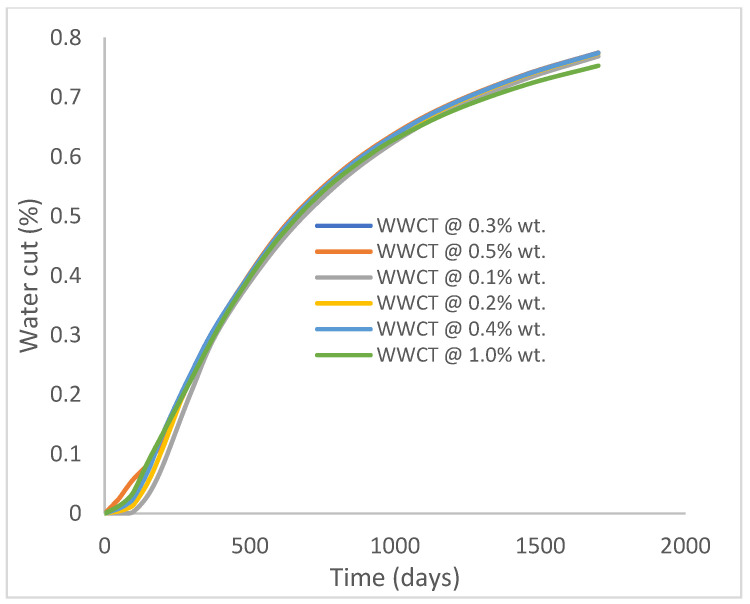
Water cut.

**Figure 31:**
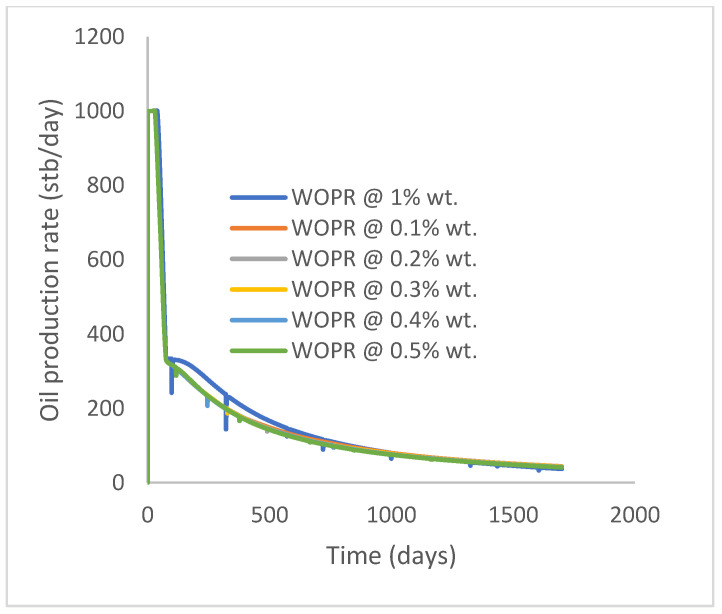
Oil production rate.

The salt contents for each polymer model are increased to 10% wt., and the results indicate a further decrease in oil recovery and production (Figure 28 and Figure 29). With reference to polymer concentrations at 1.0% wt. and 0.1% wt., oil recoveries of 40.37% and 38.17% are estimated, respectively. These results show 0.75% and 0.52% reductions in oil recovery compared to those recorded in case 3a (at a 5% wt. salt concentration). This is an indication that an increase in the salt concentrations of the polymer or the reservoir fluid will ultimately reduce oil recovery/production. Compared to case 3a, a drop in cumulative oil production is recorded at the reference polymer concentration (Figure 29). The oil production rates have dropped from 1000 stb/day to around 293 stb/day and almost declined to 41 stb/day after 1700 days. These declines and the noticeable downward spikes are a validation of the lower oil recoveries observed. Average water cuts of 75% are recorded (Figure 30), as is a drop in the oil production rate from 1000 stb/day to 315 stb/day after 100 days (Figure 31).

### 3.7. Case 4b: XG Polymer Injection at 10% wt. Salt

**Figure 32:**
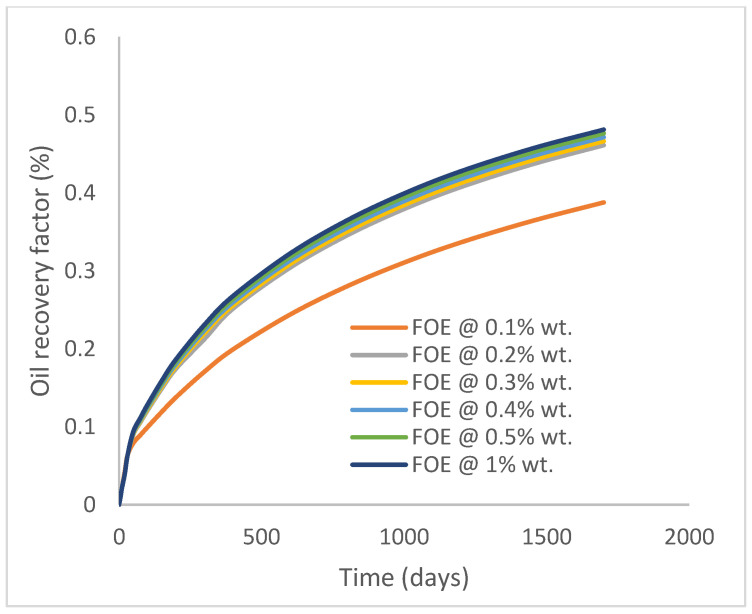
Oil recovery.

**Figure 33:**
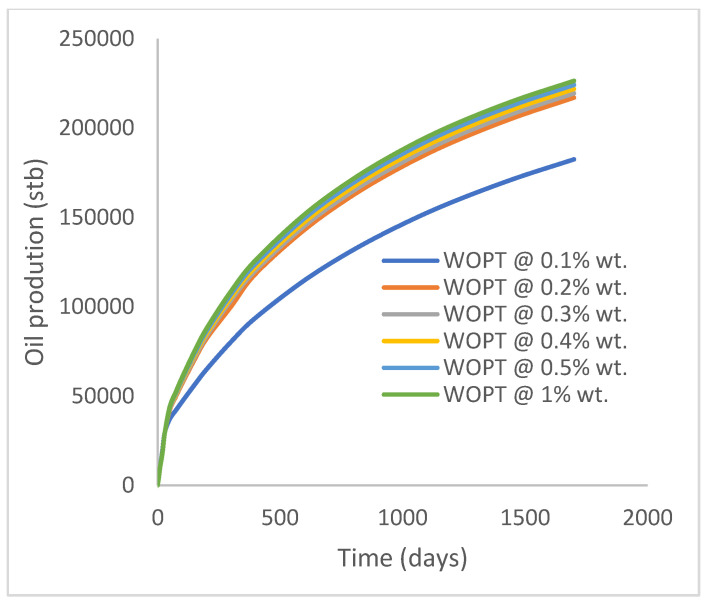
Oil production.

**Figure 34:**
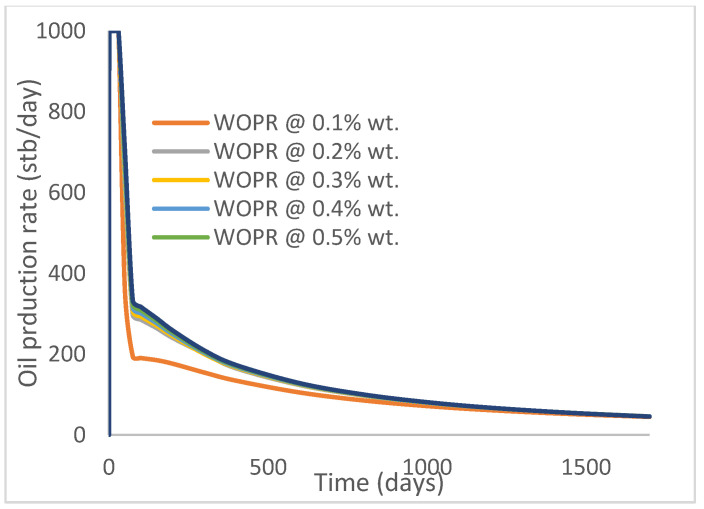
Oil production rate.

**Figure 35:**
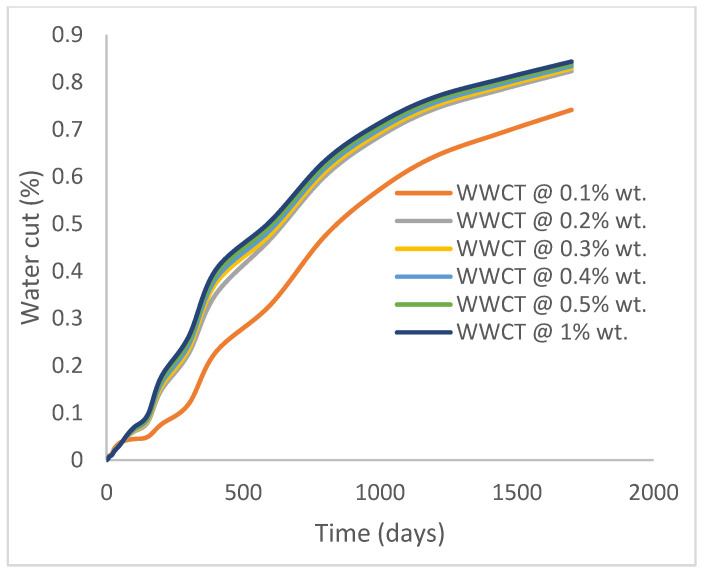
Water cut.

A further reduction in oil recovery/production is recorded in this case, like in case 4a. At a 10% salt concentration, oil recoveries of 37.78% and 48.12% are recorded during 0.1% wt. and 1.0% wt. polymer flooding, respectively (Figure 32). These estimates show reductions in oil recovery of 6.79% and 0.46% with respect to case 3b (5% wt. salt concentration) under the same polymer concentrations (5% wt. salt concentration). At the specified polymer concentrations, estimates of 182 Mstb and 226 Mstb of oil are produced. The cumulative oil produced and the production rates (Figure 33 and Figure 34) follow the trend of the reduction in oil recovery factors. The oil production rates declined after 30 days of plateau production to 190 stb/day (for 0.1% wt. polymer), and this estimate is lower than those recorded in case 3b, resulting in lower oil production. The water cut profiles are described in Figure 35 at an average value of 84%.

**Figure 36:**
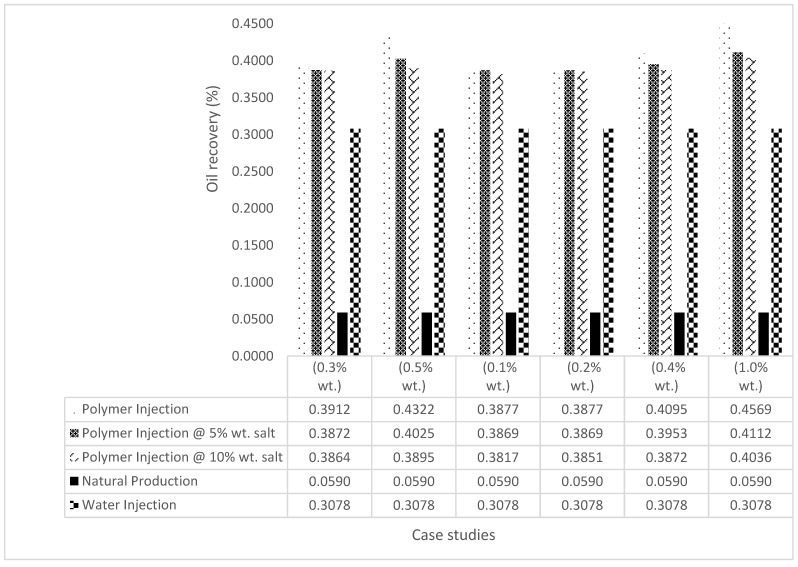
GA Oil recovery summary.

**Figure 37:**
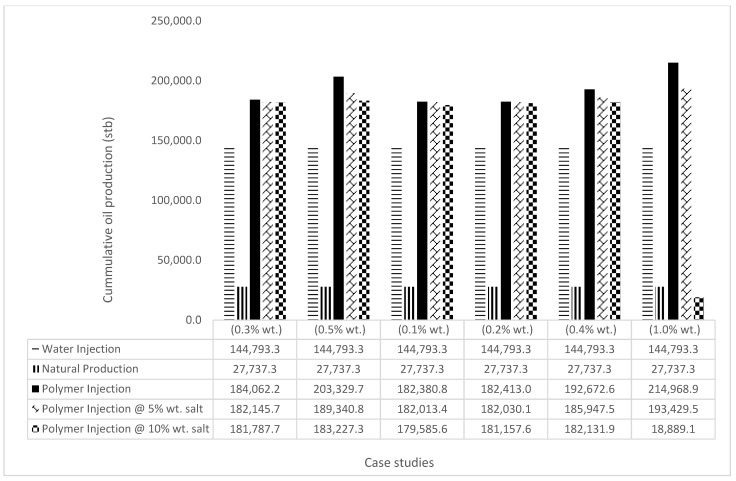
GA cumulative oil recovery.

**Figure 38:**
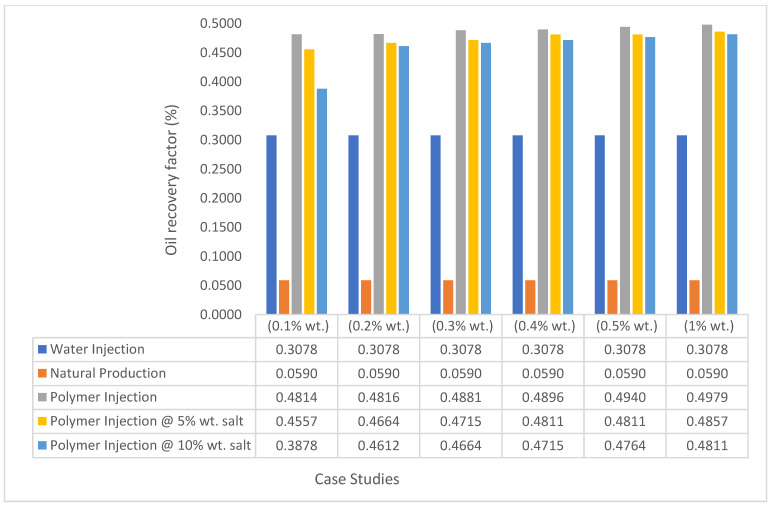
XG oil recovery summary.

**Figure 39:**
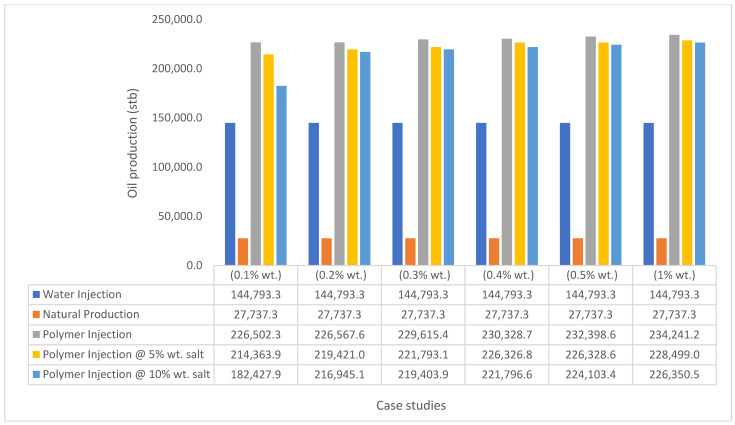
XG cumulative oil production.

The bar charts in Figure 36, Figure 37, Figure 38 and Figure 39 show the oil recovery/production profiles for each case study. As shown in the charts, the polymer flooding options increase oil recovery/production over water flooding (even with the presence of salt concentrations).

**Figure 40:**
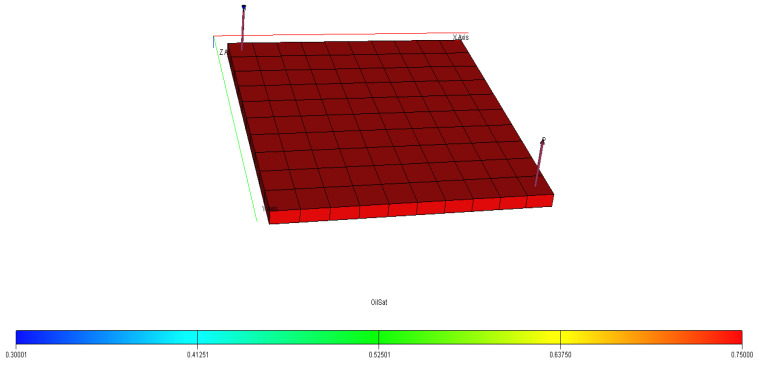
Initial oil saturation.

**Figure 41:**
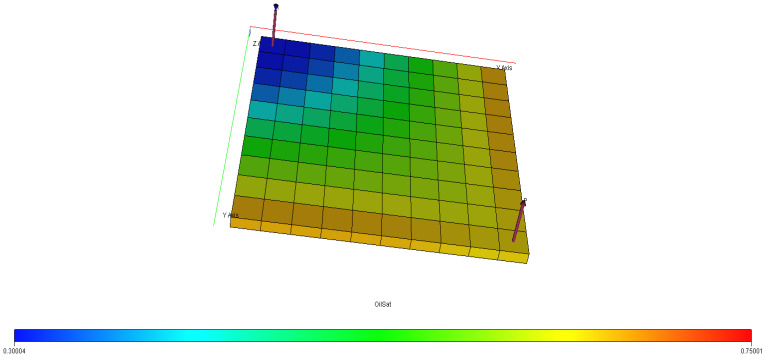
Final oil saturation after water injection.

**Figure 42:**
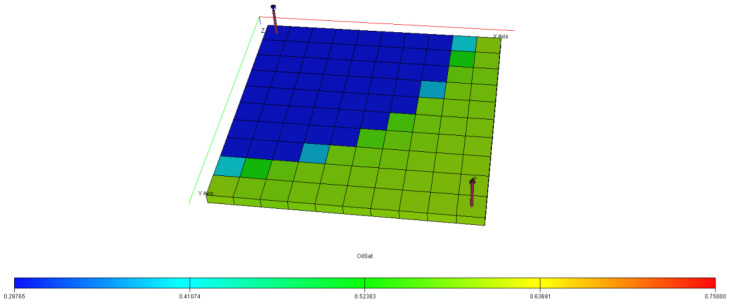
Final oil saturation after XG polymer injection of 1% wt.

**Figure 43:**
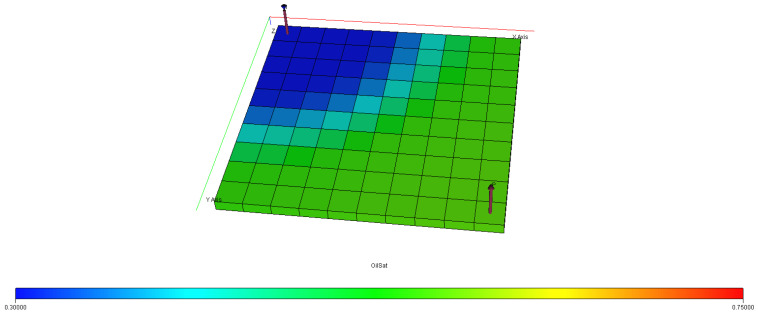
Final oil saturation after GA polymer injection at 1% wt.

**Figure 44:**
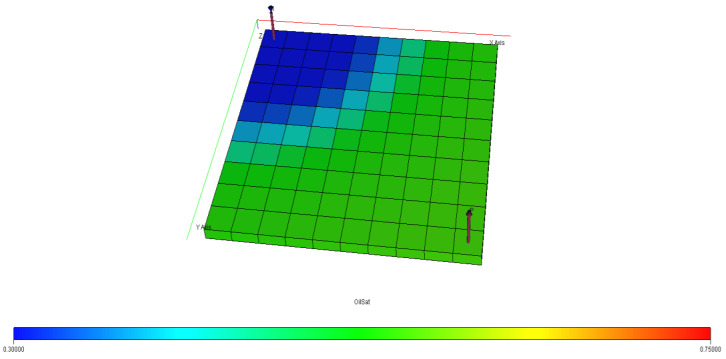
Final oil saturation after XG polymer injection at 1% wt. and 10% wt.

Figure 40, Figure 41, Figure 42, Figure 43 and Figure 44 describe the oil saturations at the initial condition, water injection, XG polymer injection (at 1% wt.), GA polymer injection (at 1% wt.), and XG polymer injection (at XG 1% wt. and NaCl 10% wt.). The red coloration on the band (to the right tail) describes regions on the model with higher oil saturations, as shown in Figure 41. During the depletion process (from natural to water injection and polymer/salt options), the rate of decrease in oil saturation is described by color saturations away from the red portion of the band. The figures show a gradual decrease in oil saturation (red coloration) to either yellow, green, or blue, signaling a reduction in oil saturation because of oil production. More oil is drained (Figure 43) during XG polymer injection at 1% wt., which substantiates the results in Figure 38.

## 4. Conclusions and Recommendations

The results have shown that increasing the polymer concentration will increase the oil recovery factor, but it is suggested that the maximum polymer concentration that will optimize oil recovery be studied, as increases show little significance on the incremental oil recovery (as observed in case 2b), to buttress the increasing adsorption rates of polymers, which may result in permeability impairment and the cost of increasing the polymer concentrations. Materials such as nanoparticles can be added to polymer mixtures to reduce the rate of their adsorption and possible reduction in permeability. The oil recoveries recorded under xanthan gum polymer flooding are higher than those observed with gum arabic because the XG had higher viscosity values at lower concentrations compared to GA, resulting in a better displacement of the heavy crude. The occurrence of higher salt concentration (10% wt.) in either polymers or reservoir fluids can reduce oil recovery during polymer flooding to 5.33% for gum arabic and 1.67% xanthan gum at 1% weight. It is also recommended that a low-salinity flooding option be considered to help reduce the salinity before the commencement of polymer flooding. This will reduce the saline content of the reservoir and reduce the adverse impact of salt on the injected polymer. It is expected that adequate water handling facilities be provided at the surface because of high water cuts.

## Figures and Tables

**Table 1:** Polymer viscosity function and salt concentration.

	PLYVISC (GA)		PLYVISC (XG)	
Polymer	Polymer Concentration, Cp (lb/stb)	Fm	Polymer Concentration, Cp (lb/stb)	Fm
A	0.1	63	0.1	165
B	0.2	83	0.2	245
C	0.3	110	0.3	440
D	0.4	130	0.4	450
E	0.5	150	0.5	540
F	1	185	1	1100

**Table 2:** PLYVICS (polymer viscosity function with salt).

PLYVISCS (XG)			PLYVISCS (GA)		
Polymer Concentration, Cp (lb/stb)	Fm @ 5% Salt	Fm @ 10% Salt	Polymer Concentration, Cp (lb/stb)	Fm @ 5% Salt	Fm @ 10% Salt
0	1	1	0	1	1
	1	1		1	1
7	20	15	3	6	2
	2	1		0.91	0.87

**Table 3:** Polymer rock property.

Dead pore spaces	0.16
Residual resistant factor	1.5
Mass rock density	1000 lb/b
Adsorption index	1
Maximum polymer adsorption value	0.005

**Table 4:** SALTVD.

Depth (ft)	Salt Con (lb/stb)	Salt Con (lb/stb)
4000	5	10

**Table 5:** Well specification.

Well Name	I Location	J Location	Group	Phase	Datum Depth (ft)
P	1	1	producer	oil	4000
I	10	10	injector	water	4000

**Table 6:** Well completion specification.

Well Name	I Location	J Location	K Upper	K Lower	Wellbore ID (ft)	Well Direction
P	1	1	1	1	1	Z
I	10	10	1	1	1	Z

**Table 7:** Well polymer/salt concentration.

Polymer Concentration, Cp (lb/stb)	0.1	0.2	0.3	0.4	0.5	1.00
WPOLYMER XG	32.6	44.31	62.78	110.41	128.72	147.21
WPOLYMER GA	11.4	14.75	21.11	36.98	42.91	49.07

## Data Availability

The data for this research will be made available upon request to the corresponding author.

## Nomenclature

FOE	Field oil efficiency/recovery
WOPT	Well oil production data
WOPR	Well oil production rate
WWCT	Well water cut
XG	Xanthan gum
GA	Gum arabic
Stb	Stock tank barrel
Lb/stb	Pounds/stock tank barrel
% wt.	Percentage weight
Kro	Relative permeability to oil
Krw	Relative permeability to water

## Appendix A

**Table A1:** Viscosity vs. shear rate profile for XG.

Shear Rate	1.0 wt%	0.5 wt%	0.4%	0.30%	0.20%	0.1 wt%
1021.38	31.95	14.95	12.45	12.05	8.65	6.05
510.69	55.3	23.2	19.8	20.9	13.7	9.3
340.46	76.95	33.75	25.95	24.75	16.35	12
170.23	137.7	53.4	42.3	47.7	26.7	18.6
102.14	209.5	82.5	67.5	58.5	35.5	24.5
51.07	389	156	119	105	58	43
10.21	1645	540	395	440	245	165

**Table A2:** Viscosity vs. shear rate profile for GA.

Shear Rate	1 wt%	0.5 wt%	0.4 wt%	0.3 wt%	0.2 wt%	0.1 wt%
1021.38	34.5	16	8.45	6.95	3.6	2.9
510.69	37	19	13.7	12.2	5.1	5.1
340.46	37.5	21	19	16.2	7.5	7.35
170.23	40.5	25	20	19	15	9.9
102.14	47.5	40	35	33	24	15.5
51.07	63	55	50	49	48	30
10.21	245	220	200	180	170	150

**Table A3:** Viscosity plot.

Wt%	XG Viscosity (cP)	GA Viscosity (cP)
0.1	165	150
0.2	245	170
0.3	440	180
0.4	450	200
0.5	540	220
1	1645	245
